# Fiber visualization for preoperative glioma assessment: Tractography versus local connectivity mapping

**DOI:** 10.1371/journal.pone.0226153

**Published:** 2019-12-12

**Authors:** Thomas Schult, Till-Karsten Hauser, Uwe Klose, Helene Hurth, Hans-Heino Ehricke

**Affiliations:** 1 Institute for Applied Computer Science, Stralsund University of Applied Sciences, Stralsund, Germany; 2 Department of Diagnostic and Interventional Neuroradiology, University Hospital Tübingen, Tübingen, Germany; 3 Department of Neurosurgery, University Hospital Tübingen, Tübingen, Germany; University of North Carolina at Chapel Hill, UNITED STATES

## Abstract

In diffusion MRI, the advent of high angular resolution diffusion imaging (HARDI) and HARDI with compressed sensing (HARDI+CS) has led to clinically practical signal acquisition techniques which allow for the assessment of white matter architecture in routine patient studies. However, the reconstruction and visualization of fiber pathways by tractography has not yet been established as a standard methodology which can easily be applied. This is due to various algorithmic problems, such as a lack of robustness, error propagation and the necessity of fine-tuning parameters depending on the clinical question. In the framework of a clinical study of glioma patients, we compare two different whole-brain tracking methods to a local connectivity mapping approach which has recently shown promising results in an adaptation to diffusion MRI. The ability of the three methods to correctly depict fiber affection is analyzed by comparing visualization results to representations of local diffusion profiles provided by orientation distribution functions (ODFs). Our results suggest that methods beyond fiber tractography, which visualize local connectedness rather than global connectivity, should be evaluated further for pre-surgical assessment of fiber affection.

## 1. Introduction

Due to improvements in diffusion-weighted magnetic resonance imaging (DW-MRI), the acquisition of high angular resolution (HARDI) datasets has become possible within clinically tolerable time frames. This has triggered the development of various signal processing and fiber reconstruction techniques which overcome the limitations of the single diffusion tensor model. Measured local diffusion profiles can be represented by, e.g., orientation distribution functions (ODFs) [1–3] or fiber orientation distribution functions (FODs) [4,5]. In these functional representations, anisotropy directions are found by detecting local maxima, allowing the reconstruction of fiber pathways by deterministic or probabilistic tractography algorithms. Additionally, local diffusion metrics, such as fractional anisotropy (FA), generalized fractional anisotropy (GFA) or mean diffusivity (MD), can be computed and depicted. Apart from color-coded FA maps, three-dimensional representations of streamlines or streamtubes are the most common visualization media used to assess fiber architecture in clinical routine.

In the preoperative assessment of white matter affection and condition near cerebral gliomas, the reconstruction and visualization of fiber pathways can help to safely maximize the extent of resection while preserving function [6,7]. The major challenges are the frequent absence of a clear normal parenchyma / tumor interface, as well as the difficulties in evaluating white matter affection in areas of peritumoral edema [8]. For this reason, the scientific literature focusses on the visualization and evaluation of white matter tracts near glioma and perilesional edema. In an evaluation study of six patients with gliomas near language-related fibers, Kuhnt et al. demonstrate the benefit of the HARDI with compressed sensing (HARDI+CS) approach over DTI [9], particularly in regions of complex fiber pathways with disrupted anisotropic diffusion. Only needing to acquire 30 gradient directions underlines the clinical practicality of their approach. Similar results were obtained in a study of eight patients with gliomas in the temporal lobe in proximity to the optic radiation (OR) [10].

Using high-definition fiber tractography (HDFT), Abhinav et al. evaluate perilesional white matter (WM) tracts in case examples of patients with glioblastoma multiforme [11]. Addressing the limitations of diffusion tensor imaging (DTI), the authors use diffusion spectrum imaging (DSI) for data acquisition, and generalized q-sampling imaging (GQI) for fiber orientation estimation. Their approach is capable of depicting perilesional pathways even in edematous zones around high-grade gliomas. However, they state that tractography studies have reproducibility issues due to their reliance on the operator’s specialized knowledge with regard to defining regions of interest (ROIs) and the segmentation of visualized tracts. Intraoperative electrical stimulation (IES) can be used to analyze the accuracy and sensitivity of fiber tracking algorithms. Bucci et al. use this method to evaluate DW-MRI tractography of corticospinal tracts in brain tumors with DTI and q-ball models, using deterministic and probabilistic methods [12]. The distances between subcortical stimulation sites and the corresponding tractography results are measured. Their results found that probabilistic tractography based on the q-ball model has the best sensitivity (79%), compared to deterministic q-ball (50%), probabilistic DTI (36%) and deterministic DTI (10%). A different approach for evaluating WM tracking is presented by Mormina et al. in a study on the qualitative and quantitative analysis of probabilistic, constrained spherical deconvolution tractography [13]. The authors examine DW-MRI data (60 gradient directions) from twenty patients with frontoparietal high-grade glioma, considering WM tract alterations of the corticospinal tract as well as the arcuate fasciculus (AF). They perform tract quantification using diffusion tensor parameters (FA, MD, linear+planar+spherical coefficients). They demonstrate that edema-affected and infiltrated tracts have lower FA values, but still preserve enough directional information for tracking algorithms to successfully track through edematous regions. However, tractography stopping criteria must be tuned regionally to, e.g., avoid false positive tracts outside edematous regions. For arcuate fasciculus reconstruction in the setting of peritumoral edema, other tractography algorithms have been used. In two-tensor, unscented Kalman filter (UKF) tractography, a diffusion model is fitted to the data during fiber tracking, taking advantage of information gained from the previous step along the fiber [14]. In [15], this method is used for surgical planning on 10 patients with left-sided tumors in the vicinity of the language-related cortex. Fiber tractography is carried out by single-tensor streamline and two-tensor UKF tractography on a diffusion-weighted dataset with 31 gradient directions. It is shown that two-tensor UKF is able to reconstruct the AF more fully than single-tensor tractography. In a study of two patients, Liao et al. focus on the performance of two-tensor UKF tractography in edema and analyze the sensitivity of tracking parameters in these regions [14]. It turns out that varying the fractional anisotropy threshold and including the free water model has a less positive effect than lowering the GFA threshold. However, FA and GFA thresholds must be individually tuned to each patient dataset. Moreover, the authors claim that whole-brain seeding or seed placement in larger regions outside the edema are the best seeding strategies. A whole-brain seeding strategy combined with UKF tractography can also be used to perform automated white matter fiber tract identification in patients with brain tumors [16]. In this study, a data-driven white matter parcellation is performed on data from healthy controls, and a fiber cluster atlas is generated using groupwise registration and spectral clustering. After key fiber tract clusters are identified in the atlas, fiber tracts from patient datasets can be identified automatically using tractography-based registration to the atlas. The results indicate that 80% of fiber clusters are identified in all 18 patients of the study. However, a major issue in tractography is the generation of false positive and false negative fibers. Furthermore, tractography still remains difficult in the vicinity of edema. In such cases, correlation with functional MRT-data can help to obtain additional, patient specific information [16]. In [17], Stadlbauer et al. examine changes in the fiber integrity, diffusivity and metabolism of the pyramidal tract adjacent to gliomas. They use quantitative diffusion tensor fiber tracking and MR spectroscopic imaging (MRSI) to examine the potential of combining both methods. Mean diffusivity, fractional anisotropy and the number of fibers per voxel (FpV) are calculated for the pyramidal tracts of the ipsilateral and contralateral hemispheres, and various metabolic concentrations are determined. As a result, quantitative DT fiber tracking shows changes in diffusivity for the pyramidal tracts of patients with sensorimotor deficits. Additionally, the use of proton MRSI can reveal whether changes in diffusivity are caused by tumor infiltration or peritumoral edema.

In an attempt to estimate the value of different tractography methods for the preoperative assessment of glioma near the motor cortex, Pujol et al. organized the “DTI Challenge”, with eight international teams applying their approaches to data from four glioma patients in order to reconstruct the pyramidal tracts [18]. The quantitative and qualitative evaluation results show a great variability between the methods. None of the approaches is able to reliably trace through edematous regions and at the same time prevent false positive tracts, e.g., in surgical cavities. As well as the frequent absence of a clear normal parenchyma / tumor interface, the dynamic interactions between neoplastic invasiveness and brain plasticity are major challenges for visualization approaches. Nevertheless, in both high-grade and low-grade gliomas, white matter tractography-based surgery is currently recognized as a valuable tool which balances the trade-off between preserving function and maximizing resection [19–23]. However, there is currently no standardized algorithmic approach for preoperative glioma assessment.

Tractography provides global connectivity depiction, but suffers from the need to individually tune parameters to patient datasets and even anatomic regions of interest. Other problems include a dependency on user interaction, e.g., for seed placement and defining inclusion/exclusion regions, a lack of robustness against noise, and error propagation during tracking. These lead to false positive and false negative tracts in the vicinity of pathological white matter [8,18]. Other visualization approaches use glyphs such as ellipsoids [24], ODF glyphs [25] or superquadric glyphs [26] to depict local diffusion characteristics, but fail to reveal global connectivity features of fiber pathways. For this reason, they have not entered into clinical routine application. Höller et al. proposed a local connectivity approach (A-Glyph LIC) as a robust fiber visualization technique which does not require user interaction and parameter tuning [27]. A-Glyph LIC is an extension of the line integral convolution (LIC) algorithm, a texture-based technique for flow field visualization originally introduced by Cabral et al. [28]. By applying a multiple-kernel LIC strategy together with the usage of anisotropic glyph samples as input patterns, the authors were able to provide color-coded LIC maps as slice images, depicting even branching and crossing fiber pathways with good contrast [29]. In an evaluation study on juvenile patients, they demonstrated the good performance of their method on different fiber pathologies, namely tumor displacement, fiber infiltration, demyelination and selective involvement of fiber tracts [27].

This paper presents a clinical evaluation study of the preoperative assessment of glioma-affected white matter with DW-MRI. We use HARDI datasets acquired from patients through routine protocols by usage of a clinical scanner, and compare deterministic and probabilistic tractography to a non-tractographic approach, namely A-Glyph LIC. We focus on the correct visualization of diffusion characteristics by the slice output images generated by the three different algorithms. In order to avoid user dependencies, whole-brain tracking with automatic seed placement is used, as proposed by [14].

## 2. Material and methods

In this clinical study of six glioma patients, who are described in Table 1, the outcome of local connectivity mapping by usage of the A-Glyph LIC algorithm and the results from deterministic and probabilistic whole-brain tractography were compared to diffusion properties, represented by ODF glyphs. High resolution T1 and T2 data, as well as HARDI datasets with 64 gradient directions, were acquired with a 3T clinical scanner. After correction of eddy current-induced distortions and subject movements as well as noise filtering, the diffusion-weighted datasets were used to calculate local diffusion profiles as ODFs. Corresponding slice images were computed from the volume datasets generated by the A-Glyph-LIC and the whole-brain tractography approaches, while high-resolution slices of visualized ODF glyphs served as a reference. Two to six regions of interest (ROIs) were selected for each patient. All slices and ROIs were selected by a neuroradiologist with long-term experience in DW-MRI. The focus was on glioma-affected regions in the white matter, particularly on peritumoral edema and tumor infiltrated tracts. In these regions judgement of fiber integrity is of vital interest in surgery planning, to balance the trade-off between preserving function and maximizing resection.

**Table 1:** List of patients used for the study and their pathologies and symptoms.

	Gender, age	Pathology	Symptoms
Patient A	Female, 54 years	Glioblastoma in the right frontal lobe	Headache, concentration deficits
Patient B	Female, 22 years	Cavernoma in the white matter of the right parietal lobe	Seizures
Patient C	Female, 34 years	Diffuse astrocytoma WHO II of the left frontal lobe	Symptomatic epilepsy
Patient D	Male, 57 years	Left parietal glioblastoma	Aphasia
Patient E	Female, 58 years	Metastasis of adenocarcinoma in the left basal ganglia	Seizures
Patient F	Male, 76 years	Glioblastoma in the right precentral gyrus	Seizures

### Data acquisition

Six patients suffering from low- or high-grade glioma were selected for this study. Their images were acquired as part of ongoing research studies, approved by the ethics committee of the medical faculty of the Eberhard Karls University of Tübingen. Informed written consent was obtained from the patients.

All patient datasets were acquired with a 3T MRI scanner (Siemens Skyra, Siemens Healthineers, Erlangen, Germany) at the University Hospital Tübingen. Table 2 gives an overview of the acquisition protocols used.

**Table 2:** Overview of the clinical acquisition protocols.

Sequence	DW-MRI Sequence	T1 Sequence 1	T1 Sequence 2	T2 Sequence 1	T2 Sequence 2
**TR/TE [ms]**	6100/85	2300/2.32	2300/3.51	3200/408	5000/387
**Matrix**	114x114x50	208x256x256	176x256x256	208x512x512	208x512x512
**Voxel length [mm]**	2.0x2.0x2.0	0.9x0.9x0.9	1.0x1.0x1.0	0.9x0.47x0.47	0.9x0.47x0.47
**DWI directions**	64				
**B-Value [s/mm^2^]**	1000				
**Used for**	A–F	A, C, D	B, E, F	A	C

### Data preprocessing

All datasets went through the same preprocessing procedures. First, using the FMRIB Software Library (FSL, Analysis Group, FMRIB, Oxford, UK), a brain mask was generated by applying an automatically determined FA threshold [30, 31]. The MRtrix software package (Brain Research Institute, Melbourne, Australia) masking algorithm was used for the probabilistic tractography approach. All brain masks were visually inspected and any errors, e.g., holes, which may occur especially in edematous regions or inside gliomas, were manually corrected.

FSL was used to correct eddy current-induced distortions and patient movements during acquisition, using a mutual information-based, retrospective motion correction scheme [32].

**Fig 1:**
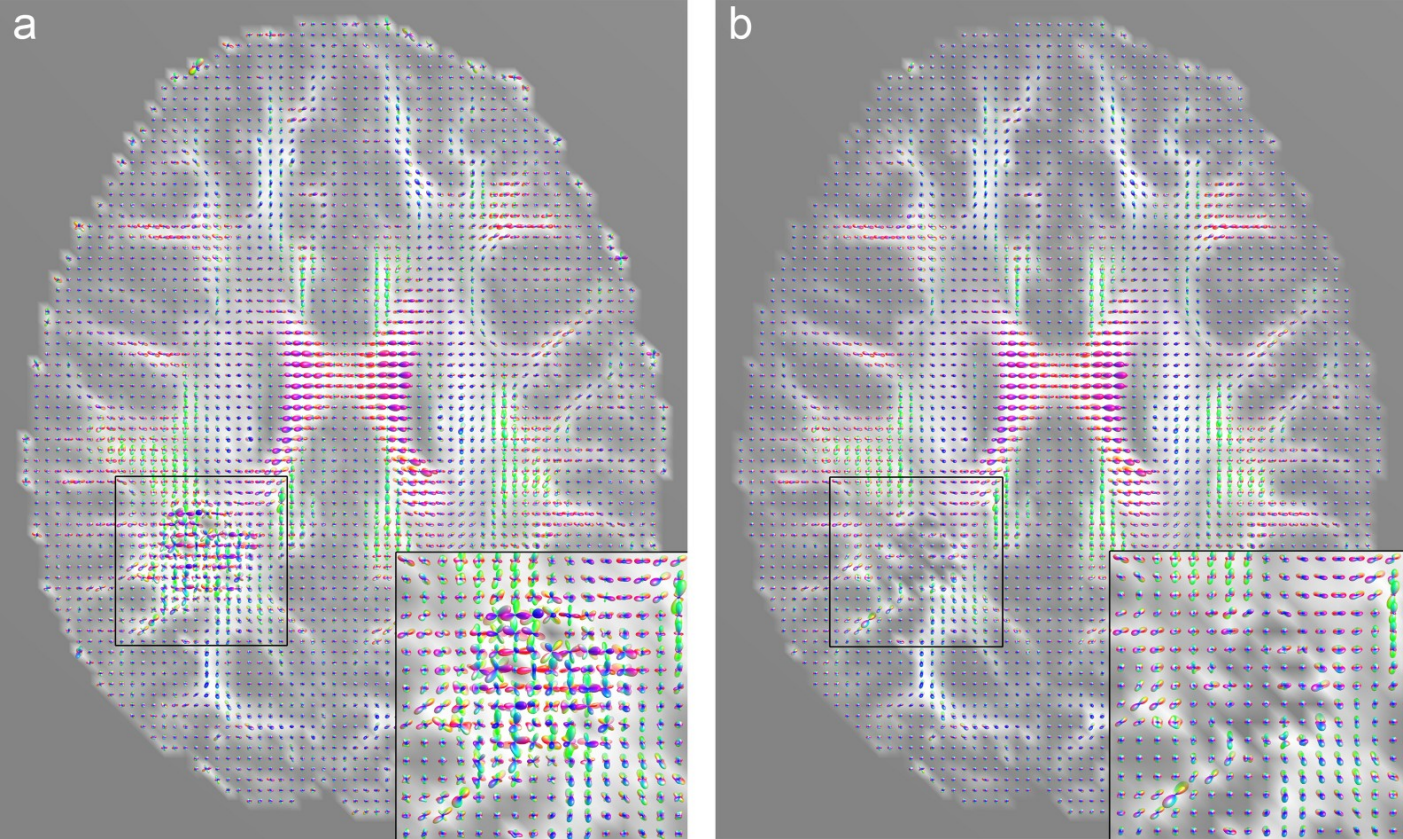
Effect of denoising DW-MRI data from patient B on CSA-ODFs. To provide anatomic context, GFA slice images are used as the background for the CSA-ODF glyphs. Images (a) and (b) both show the same slice, calculated with Matlab. The marked and enlarged areas show the tumor region inside the right hemisphere. (a) CSA-ODFs calculated from noisy DW-MRI data. (b) CSA-ODFs calculated after application of the OLPCA noise reduction scheme. Noise reduction was applied to the entire DW-MRI dataset but is most effective in regions with a low SNR, such as the marked and enlarged tumor region.

When acquired under clinical conditions, DW-MRI datasets often suffer from a low signal-to-noise ratio (SNR) and are thus subject to misinterpretations and visualization errors [33]. Therefore, we applied a noise filtering procedure to the original diffusion data using the overcomplete local principal component analysis (OLPCA) approach described by Manjón et al. [34]. For this we used a Matlab implementation (MathWorks Inc., Natick, Massachusetts, USA) made available by the author [35]. Denoising the data is most effective in regions with a low SNR and allows, amongst other things, the stable computation of constant solid angle ODFs (CSA-ODFs), which tend to degenerate in regions of low signal and high noise. Fig 1 illustrates the effect of denoising the diffusion data on an axial slice image from patient B. When computed from noisy data (Fig 1A), particularly in the zoomed regions, the CSA-ODFs are degenerated and show a high level of anisotropy, which is in contrast to the underlying signal intensities of the B0-image and the gradient-specific diffusion images. After data denoising, the CSA-ODFs have reasonable sizes and show low anisotropy (Fig 1B), consistent with the results of computing the ODFs with the method of Descoteaux et al. [2].

### ODF computation

The orientation distribution function is a model-free functional representation of the local diffusion profile. ODF reconstruction can be performed by applying a Funk-Radon transform (FRT), using either spherical radial basis functions (sRBF) [1] or spherical harmonics [2,36]. In order to sharpen the profiles, a normalization step is necessary, which in the presence of artifacts and outliers is not always straightforward. Alternatively, the constant solid angle (CSA) ODF can be computed with the method proposed by Aganj et al. [3]. This includes an intrinsic normalization and leads to ODFs with realistic sharpness, allowing easy detection of anisotropic diffusion directions. Even more sharpness is provided by the fiber orientation distribution (FOD), which is based on the constrained spherical deconvolution (CSD) algorithm [4,5]. This applies signal deconvolution with a kernel, modeling a single coherently-orientated fiber bundle. This kernel can be estimated from the individual HARDI dataset by manual or automatic procedures, but introduces modeling assumptions. It has been demonstrated that by the CSD approach a high angular resolution may be provided, allowing fiber directions to be extracted, even in regions with acute-angled fiber crossings. However, by usage of a single-fiber model, modeling assumptions are introduced. As an alternative, the CSA approach to ODF computation is completely model-free and also leads to quite sharp diffusion profiles [3] with a good angular resolution, resolving fiber crossings at about 45° [37]. Thus, it outperforms the ODF computation method originally proposed by Tuch [1,38], and allows fiber directions to be determined easier. As explained above, a Matlab implementation, made available by Aganj [39], was used. From this, we were able to produce high-resolution slice images of the CSA-ODF glyphs, corresponding to planar slices from the A-Glyph LIC and the probabilistic/deterministic tractography volumes. CSA-ODF glyphs are constructed by deforming the surface of a sphere according to ODF values distributed over a half sphere. After a directional color-coding scheme [40] is applied, the glyphs depict local anisotropy directions.

We used a spherical harmonic basis order of 4 and a regularization parameter of 0.15 to generate the CSA-ODFs. With these parameters, ODF glyphs with sufficient sharpness and a shape consistent with the ODF glyphs computed with the method of Descoteaux could reliably be provided for all patient datasets. In order to provide anatomic context, we calculated the generalized fractional anisotropy values from the CSA-ODFs [1] and used planar GFA-slice images as a background for the ODF glyph visualizations.

### Local connectivity mapping by line integral convolution with anisotropic glyph samples (A-Glyph LIC)

Different to tractography, local connectivity mapping approaches visualize fiber connectivity by analysis of diffusion profiles in a voxel’s immediate neighborhood. The A-Glyph LIC approach used here is an extension of the line integral convolution algorithm originally introduced by Cabral et al. [28]. The basic principles and steps of the A-Glyph LIC method have already been described by Höller et al. in [29]. Therefore, we give only a short summary of the algorithm’s most important steps here. First, a high-resolution input pattern is generated with a voxel size of 0.1 mm. The input pattern is filled with multi-cylindrical glyph samples, which are placed along very short streamlines, tracked locally within the interpolated diffusion data. Local tracking distances are of the order of the voxel size of the original diffusion dataset. The seeds are placed randomly throughout the whole high-resolution dataset. Instead of FODs, as in Höller’s method, we use CSA-ODFs to encode local anisotropy characteristics by scaling the cylindrical glyphs with the length of the CSA-ODF maxima. Thus, the high-resolution anisotropic glyph pattern generated consists of cylindrical glyphs which indicate local anisotropy directions as well as extent. In the next step, the pattern is used as an input for the multiple kernel LIC algorithm. This algorithm smooths the input-pattern along a kernel, following the global maxima of the CSA-ODFs as well as the second local CSA-ODF maxima, which is found using a Newton-Raphson gradient ascent algorithm. The two-dimensional smoothing kernel allows the depiction of crossing and branching fibers. The kernel is generated by tracking locally over very few steps of the order of the resolution of the original diffusion dataset, which is about 2 mm. The resulting 3D LIC volume is directionally color-coded and visualized by planar slice images of 1 mm thickness. To incorporate additional anisotropy information, the pixel lightness of A-Glyph LIC slice images is scaled by the GFA value calculated from the CSA-ODFs. Additionally, the LIC slices are fused with T1 or T2 images to provide anatomic context. We used the fiberViewMR software package (Stralsund University, Stralsund, Germany) [41] for A-Glyph LIC processing.

### Probabilistic streamline tractography

Deterministic tracking approaches often suffer from false negative fiber reconstructions, missing fiber structures which are highly relevant for surgery planning. Probabilistic approaches may improve tracking through regions of low anisotropy or with less distinct directions. These areas are of vital interest in preoperative glioma assessment. For this reason, probabilistic streamline tractography was used as a promising and established reference method. The MRtrix software package (Brain Research Institute, Melbourne, Australia) used, implements probabilistic tracking on the basis of a first-order integration over FODs (iFOD1). This tracking algorithm is referred to as “SD_PROB” in [42]. To avoid depending on the user’s skills in seed region definition and to allow reproducibility, a whole-brain tracking was applied with seeds generated at random by uniform sampling of the white matter mask [42]. Following the recommendations in [16,43], experiments with 50,000, 75,000 and 100,000 tracts to be generated were carried out. For all the diffusion datasets investigated, the most reasonable results were achieved with 75,000 tracts. To allow tracking through low anisotropy regions, e.g., edema, an FOD amplitude of 0.1 was set as the tract stopping threshold. For all other parameters, defaults provided by MRtrix were chosen, e.g., stepsize of 0.2 mm, minimum radius of curvature of 1.0 mm, minimum track length of 10 mm, and maximum track length of 200 mm. From the resulting streamline volumes, slice images were generated by clipping to slabs of 1.0 mm thickness, corresponding to A-Glyph LIC slices in orientation and position. As an anatomic reference, the tractography slices were fused with T1 slice images.

### Deterministic streamline tractography

Deterministic tracking approaches based on HARDI datasets may lead to good results, even in regions of crossing and branching pathways, if the underlying diffusion profiles are adequately represented by sharp ODFs or FODs [42]. To perform deterministic streamline tractography, a fiber assignment by continuous tracking (FACT) algorithm [44] based on CSA-ODFs was used. This algorithm was implemented through the modular software platform OpenPDT, which was developed by our group. In order to keep results independent from the operator’s skill in correctly placing seeds and to avoid inter-operator variability, whole-brain tractography [14] was applied with seed points defined randomly in continuous space covering the whole brain [45]. To be consistent with the probabilistic tractography, 75,000 tracts were calculated for each patient dataset, with a step size of 0.2 mm, a minimum GFA value of 0.1 and a minimum curvature angle of 30°/mm. Only tracts with a minimum length of 10 mm and a maximum length of 200 mm were accepted. To compare the results of the deterministic streamline tractography with those from the probabilistic tractography and the A-Glyph LIC algorithm, the resulting streamline volume was clipped to slabs of equal position, thickness and orientation. Additionally, all slices were fused with T1 slice images.

### Evaluation study

This study focusses on the question of whether an LIC-based visualization method is capable of depicting tumorous fiber affections in a way that is consistent with local diffusion profiles represented by CSA-ODFs. In order to compare its performance with more established methods, its performance is compared to that of deterministic and probabilistic streamline tractography algorithms. The study protocol includes a data preparation step, in which a neuroradiologist with long-term experience in clinical MRI views multi-planar reconstructions of the acquired T1 and T2 patient datasets and interactively marks regions of interest (ROIs) with ellipsoidal shape in the vicinity of the lesion. For each T1 or T2 image containing a ROI, four corresponding planar slices with the same thickness, orientation and position were generated, depicting:

- CSA-ODF glyphs with ROI overlay fused with a GFA image,
- directionally color-encoded A-Glyph LIC with ROI overlay fused with T1,
- directionally color-encoded streamlines from deterministic, whole-brain tractography with ROI overlay fused with T1, and
- directionally color-encoded streamlines from probabilistic, whole-brain tractography with ROI overlay fused with T1.

A total of 18 ROIs were defined and analyzed. The slice thickness of all images was set to 1.0 mm. The slice images with the CSA-ODF glyphs (a) served as a reference and the visualization results in images b)—d) were visually inspected for consistency.

## 3. Results

**Fig 2:**
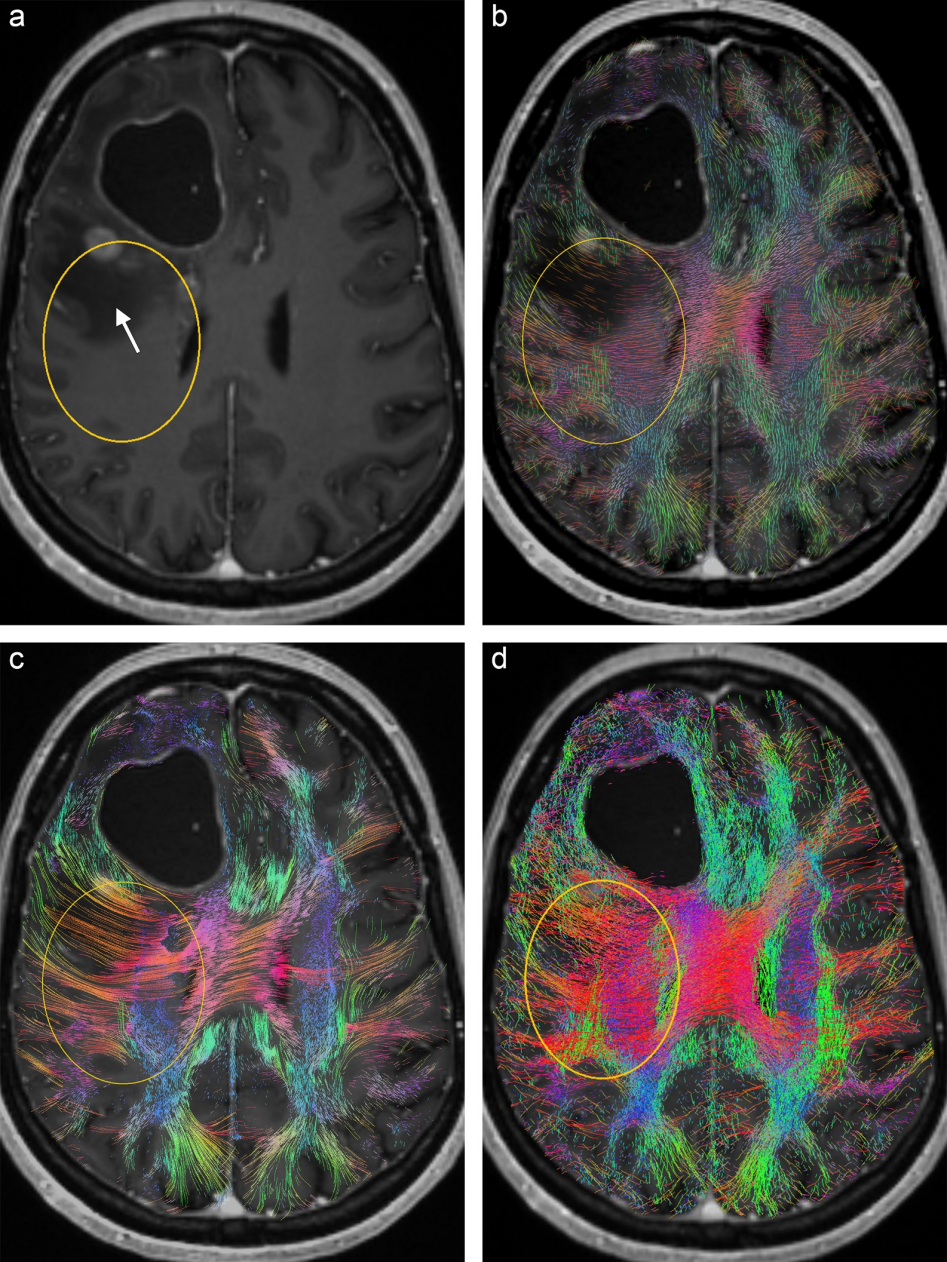
Axial slice from patient A: (a) T1 with ROI (yellow) which includes an edema/non-enhancing tumor (arrow), (b) A-Glyph LIC fused with T1, (c), deterministic tractography result fused with T1, and (d) probabilistic tractography result fused with T1.

In many clinical studies related to fiber visualization from diffusion MRI data, visualization errors and the reliability and robustness of the algorithms used, have been the main subject of interest. The clinical focus of the evaluation study presented in this paper, is on the affectedness of white matter tracts in the vicinity of brain tumors, particularly near gliomas and within perilesional edema. With regard to erroneous visualizations, a distinction between false positive and false negative tracts is made. The most important findings are listed by Table 3. Erroneous visualizations of tracts, which are generated in regions with non-anisotropic voxels, are classified as false positives. Non-anisotropic voxels are characterized by CSA-ODF glyphs with a nearly perfect spherical shape. In some cases the classification of non-isotropic regions could be confirmed by a look at corresponding T1- and T2-weighted slice images, e.g., by delineation of necrotic areas. False negatives refer to tracts, which are not or not adequately depicted, although there is enough anisotropy in the corresponding region. In these cases the CSA-ODF glyphs show bulges which can be identified as distinct deviations from a spherical shape. False positive tracts are generated by both, deterministic as well as probabilistic tractography, in regions of perilesional edema (patient A, Fig 2), low grade glioma (patient C, Fig 5) and the periphery of grade IV glioma (patient E, Fig 9). Deterministic tractography also shows false positives in fiber crossings near a glioblastoma (patient F, Fig 11). False negative visualizations are produced by probabilistic tractography in the peripheral region of a cavernoma (patient B, Fig 4) and by A-Glyph LIC in a region of pyramidal tracts running orthogonally to the slice image plane (patient A, Fig 2).

**Table 3:** List of findings (false positives and negatives) and their relation to the three visualization methods.

Finding	Method	Region/Pathology	Patient
**False positive tracts**	det./prob. Tr.	perilesional edema	A
	det./prob. Tr.	low grade glioma	C
	det./prob. Tr.	grade IV glioma (periphery)	E
	det. Tr.	fiber crossing near glioblastoma	F
**False negative tracts**	prob. Tr.	cavernoma (periphery)	B
	A-Glyph LIC	fibers running orthogonal to image plane	A

**Fig 3:**
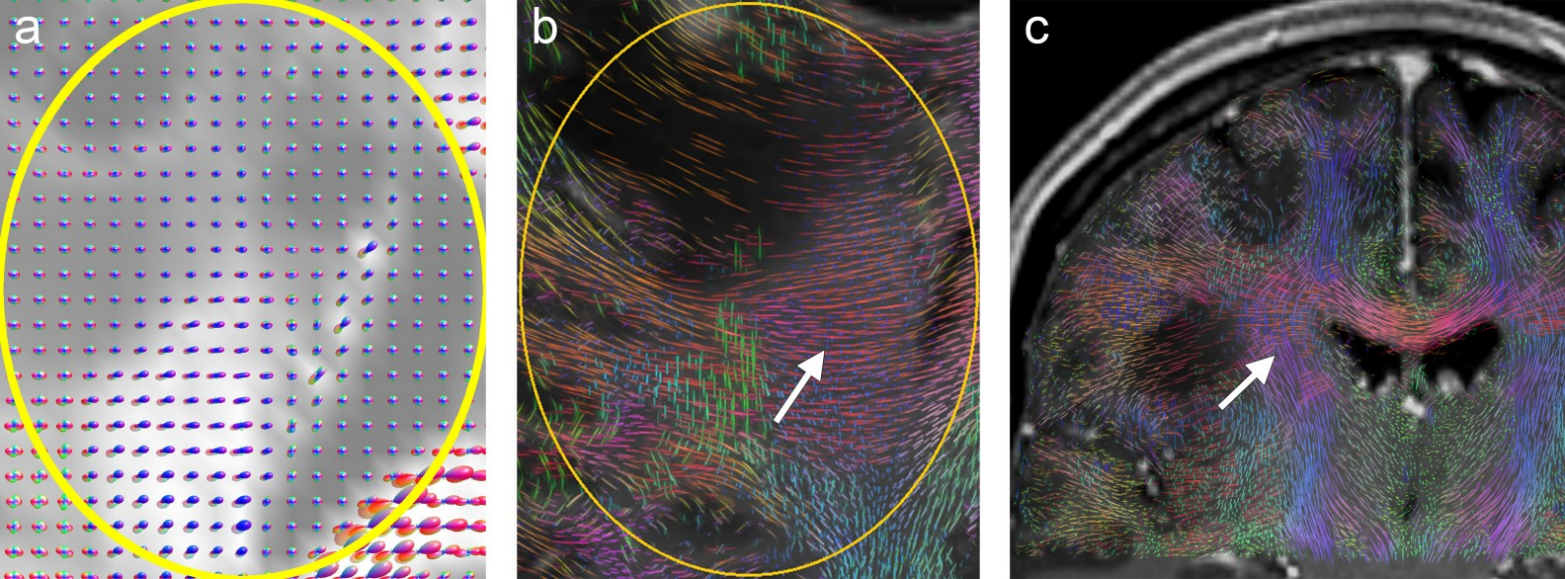
(a) CSA-ODF glyphs in ROI of Fig 2 with GFA slice as background, (b) zoomed A-Glyph LIC image, and (c) coronal A-Glyph LIC slice.

A more detailed explanation of these findings as well as a discussion of positive results are presented by the following paragraphs. Fig 2 shows the results from patient A, with the corresponding axial slice images: (a) T1-weighted image with oval ROI (yellow) containing edematous tissue and nonenhancing tumor tissue in its upper part (arrow), (b) A-Glyph LIC image, (c) results from deterministic tractography, and (d) slice from probabilistic tractography volume. Differences can be seen between the results of the three visualization methods: whereas the three methods correctly depict the absence of fibers in the necrotic core of the tumor in the frontal lobe, the results differ in the region affected by edema. Both tractography methods do not depict any fiber rarefication in the edema, while the A-Glyph LIC result suggests a noticeable loss of anisotropy. This is supported by the CSA-ODF glyphs (Fig 3A), which exhibit an almost spherical shape in this region, thus indicating isotropic diffusion. The A-Glyph LIC algorithm seems to fail to depict pyramidal fibers (blue) running orthogonally to the selected slice image plane. By zooming the ROI, these fibers are better visualized by blue dots (arrow in Fig 3B), which in the overview image are optically obscured by the red fiber structures. In a corresponding coronal A-Glyph LIC plane pyramidal fibers are more effectively visualized (Fig 3C).

**Fig 4:**
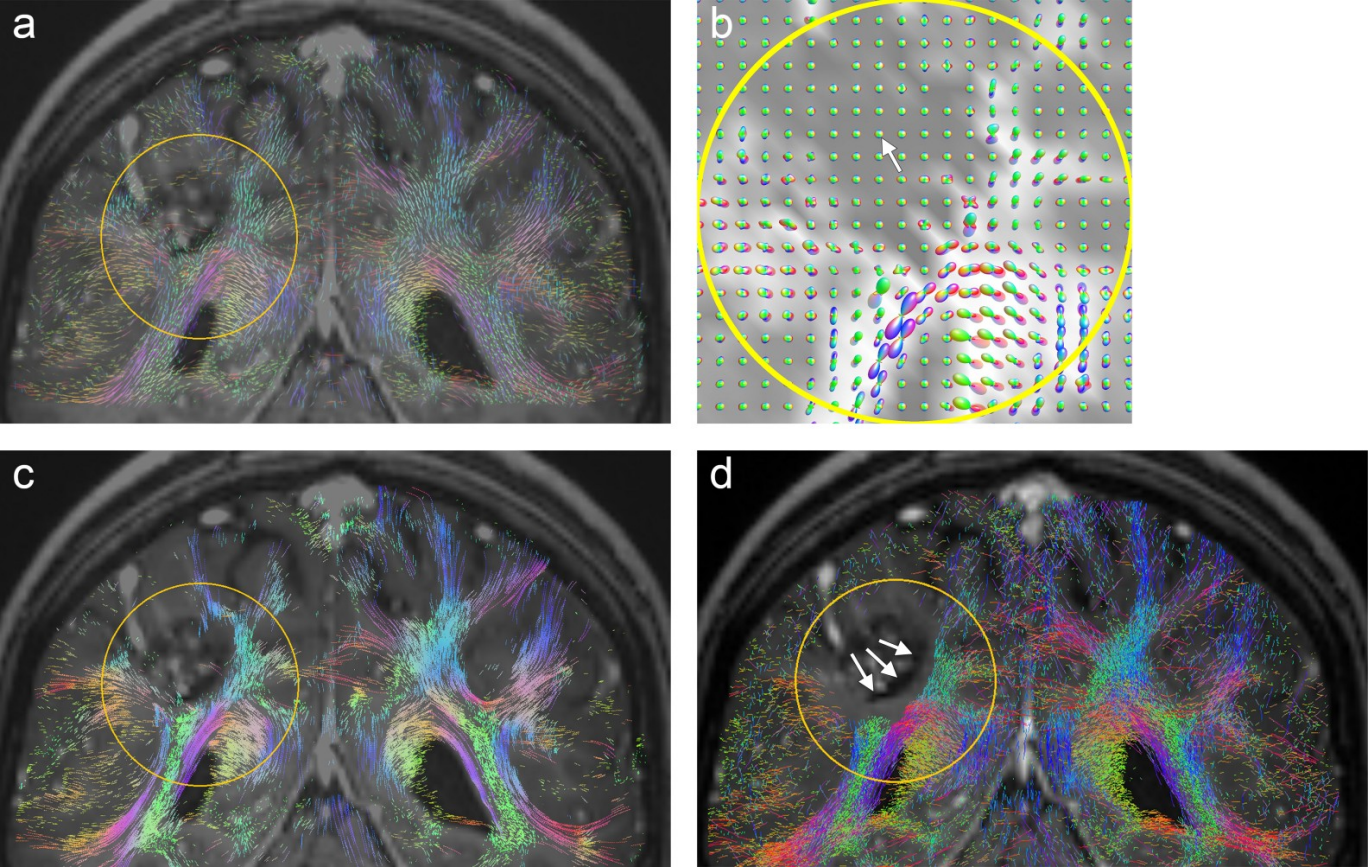
**Visualization results from patient B: (a) Coronal A-Glyph LIC slice with ROI (yellow), (b) ODF glyphs, (c) corresponding slices from tractography results, (c) deterministic, and (d) probabilistic.** Arrows in (d) indicate unrealistic fiber disruptions in the vicinity of the lesion.

Fig 4 illustrates the results from patient B. The central contrast-enhancing part of the tumor is visualized free of fibers by all three methods (Fig 4A, 4C and 4D). This is supported by the ODF glyphs, which have an almost perfectly spherical shape in this area (arrow in Fig 4B). However, unlike deterministic tractography and A-Glyph LIC, probabilistic tractography shows the disruption of fibers in a circular belt surrounding the tumor (arrows in Fig 4D). Both, deterministic as well as probabilistic tractography employ a stopping criterion of 0.1 in terms of GFA and FOD amplitude, respectively. While ODF glyphs indicate residual anisotropy, FODs in this region seem to suggest no anisotropy (see Fig 12A), which induces probabilistic tractography to stop. If the postulated FOD length parameter was further reduced, the probabilistic tractography approach might also depict fibers in the immediate tumor neighborhood.

**Fig 5:**
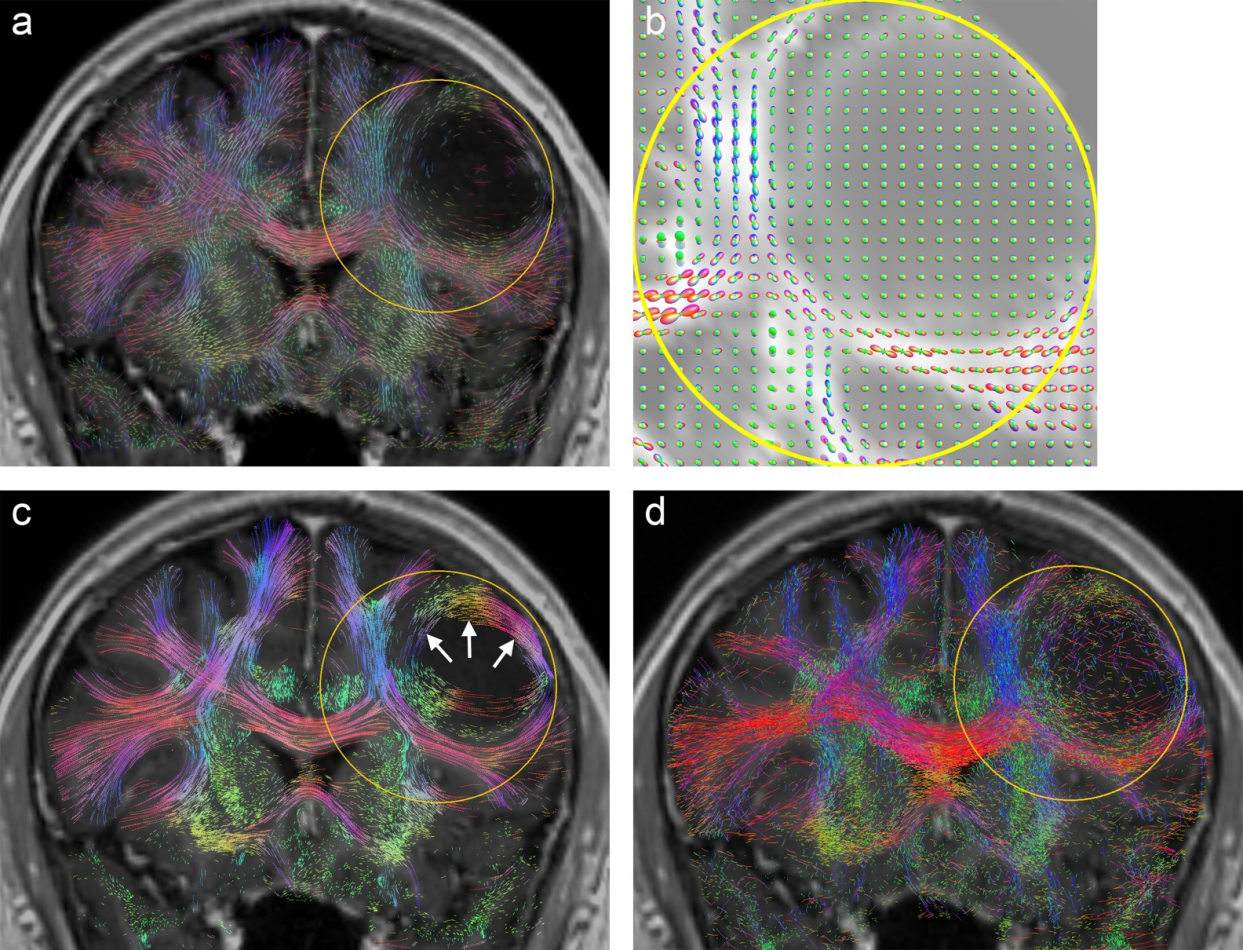
**Visualization results from patient C: (a) Coronal A-Glyph LIC slice with ROI (yellow), (b) ODF glyphs, corresponding slices from deterministic (c) and probabilistic (d) tractography results.** Arrows in (c) indicate an unrealistic belt of fibers surrounding the core of the lesion.

Fig 5 also emphasizes the differences between the three methods with images from patient C. The ODF glyphs inside the central part of the low grade glioma in the ROI are small, and show only a small amount of anisotropy (Fig 5B). The A-Glyph LIC result reflects these findings in part: some residual anisotropy is depicted (Fig 5A). With probabilistic tractography, a larger number of fibers are visualized inside this region, but fewer than in the contralateral region (Fig 5D). In this example, the whole-brain probabilistic tractography approach tends to generate false positive fibers. Deterministic tractography shows a belt of circular fibers surrounding the cavernous region (arrows in Fig 5C), which is not consistent with other clinical findings in T1 and T2 images, as well as anisotropy features, which are depicted by the ODF glyphs.

**Fig 6:**
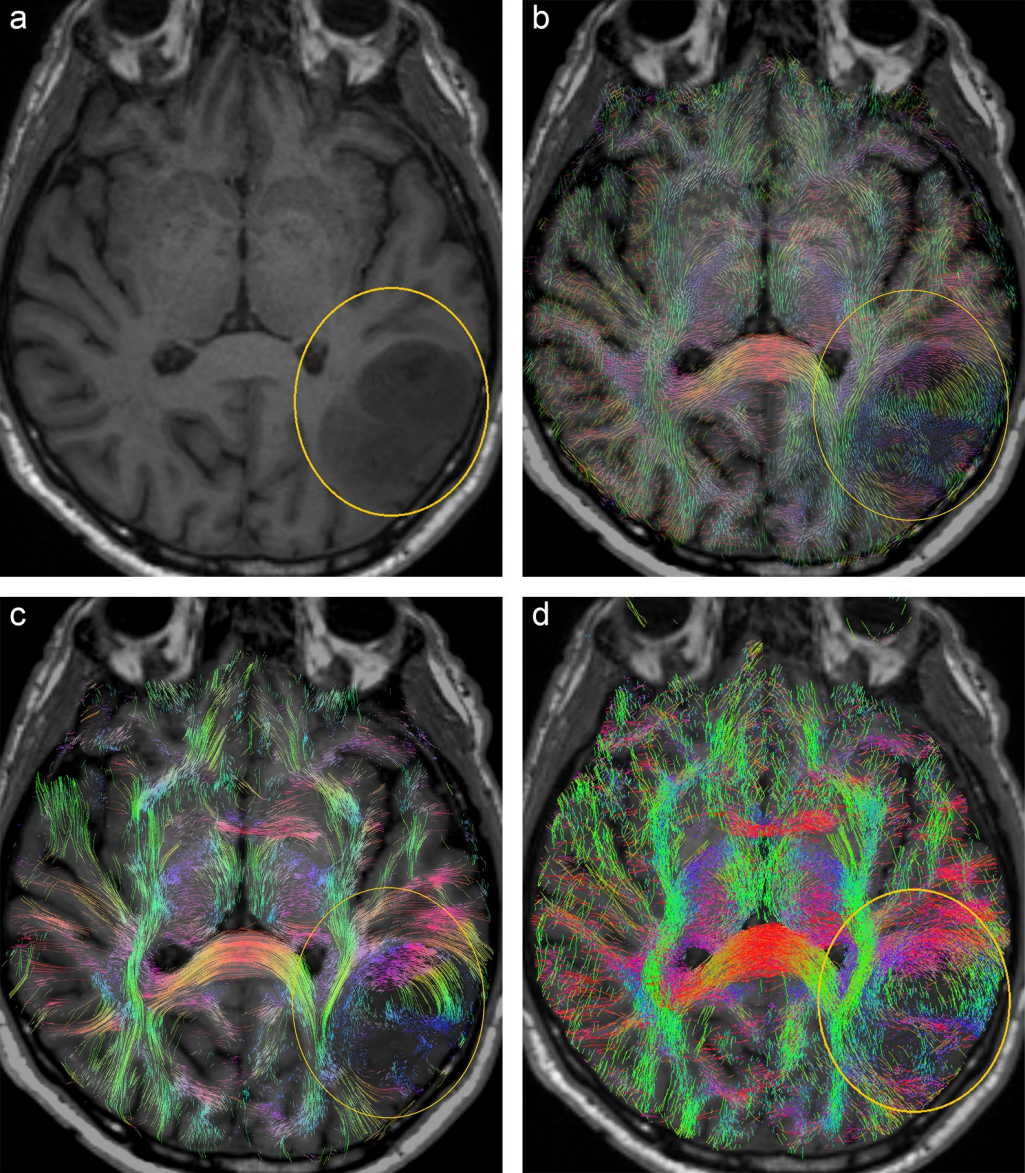
Visualization results from patient D (transaxial slices): (a) T1 image with ROI (yellow), corresponding slices from A-Glyph LIC (b), deterministic (c) and probabilistic (d) tractography.

**Fig 7:**
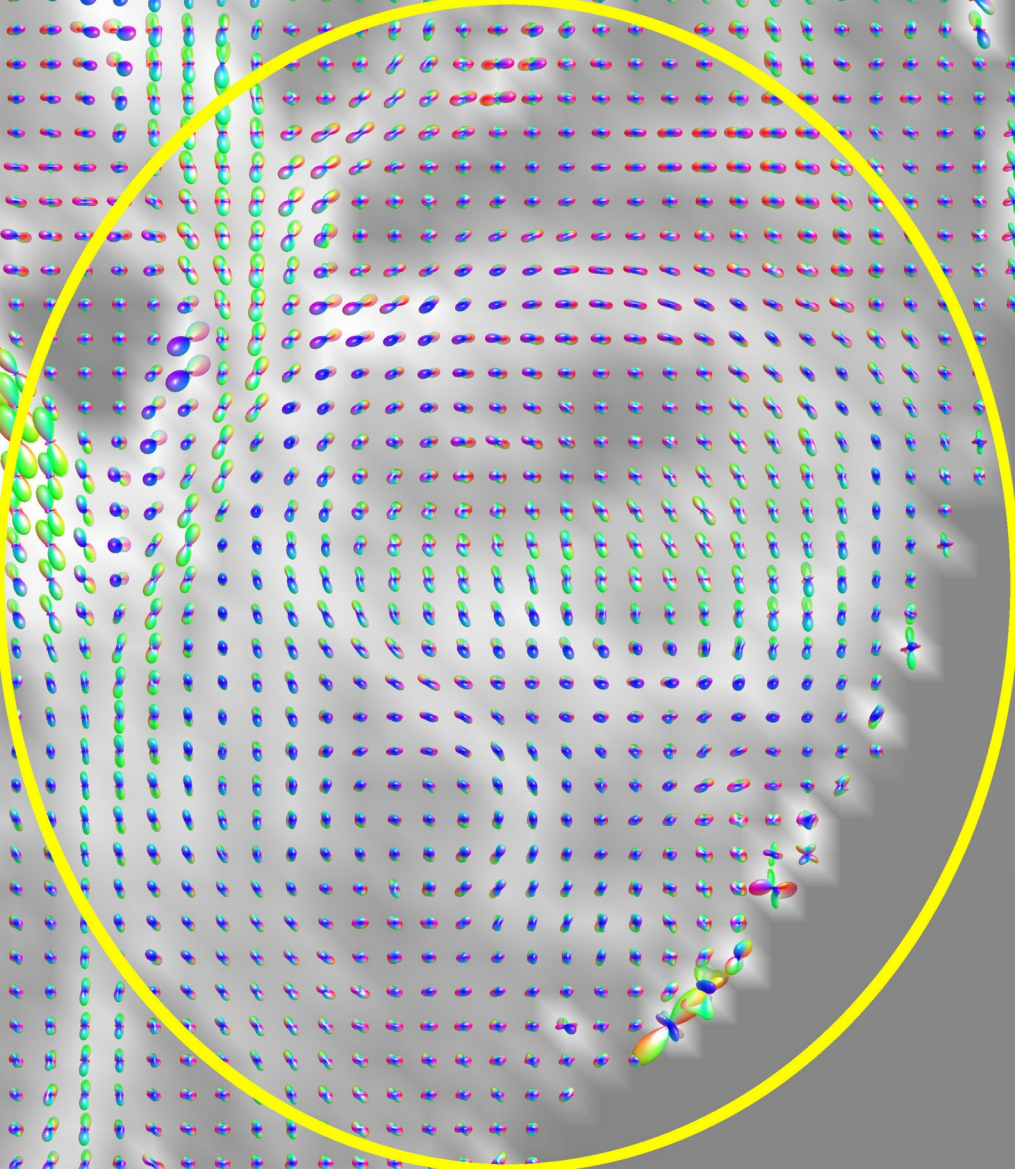
CSA-ODF glyphs in ROI from Fig 6.

Results from patient D show similar fiber visualizations for the three methods (Fig 6). However, the fibers within the tumor region are not consistent with what is expected from a clinical point of view and from the T1 images. A distinct reduction of anisotropy and fiber density, anticipated in the tumor region (yellow), is not depicted by the three methods (Fig 6B, 6C and 6D). The shapes of the CSA-ODFs reveal that the visualizations are at least consistent with the underlying diffusion data, which indicate sufficient anisotropic diffusion profiles in the region of interest (Fig 7). In this case, the heterogeneity of the lesion seems to include the remaining fiber tracts.

**Fig 8:**
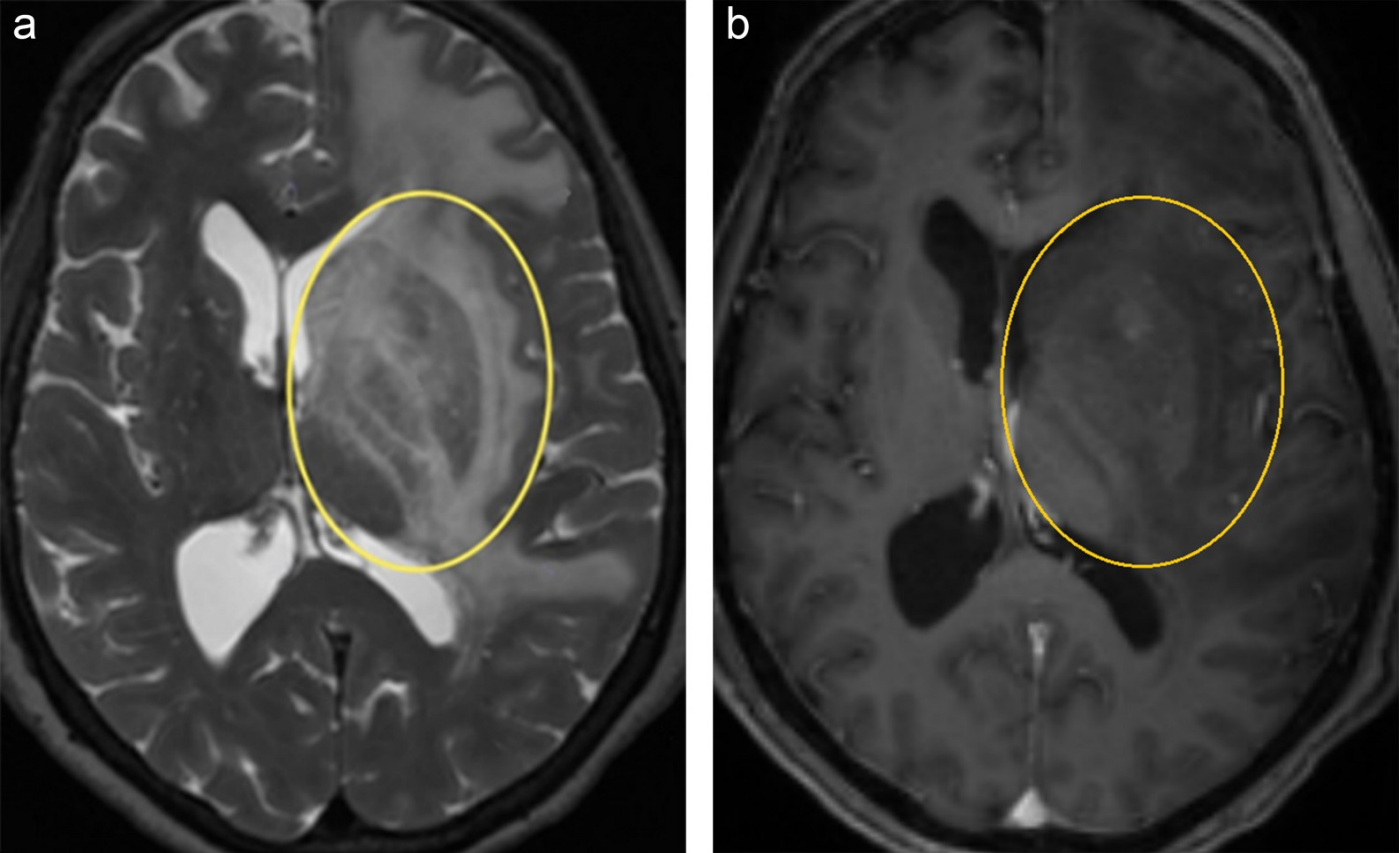
Transaxial slices from patient E: (a) T2 image with ROI (yellow), (b) corresponding T1 slice.

**Fig 9:**
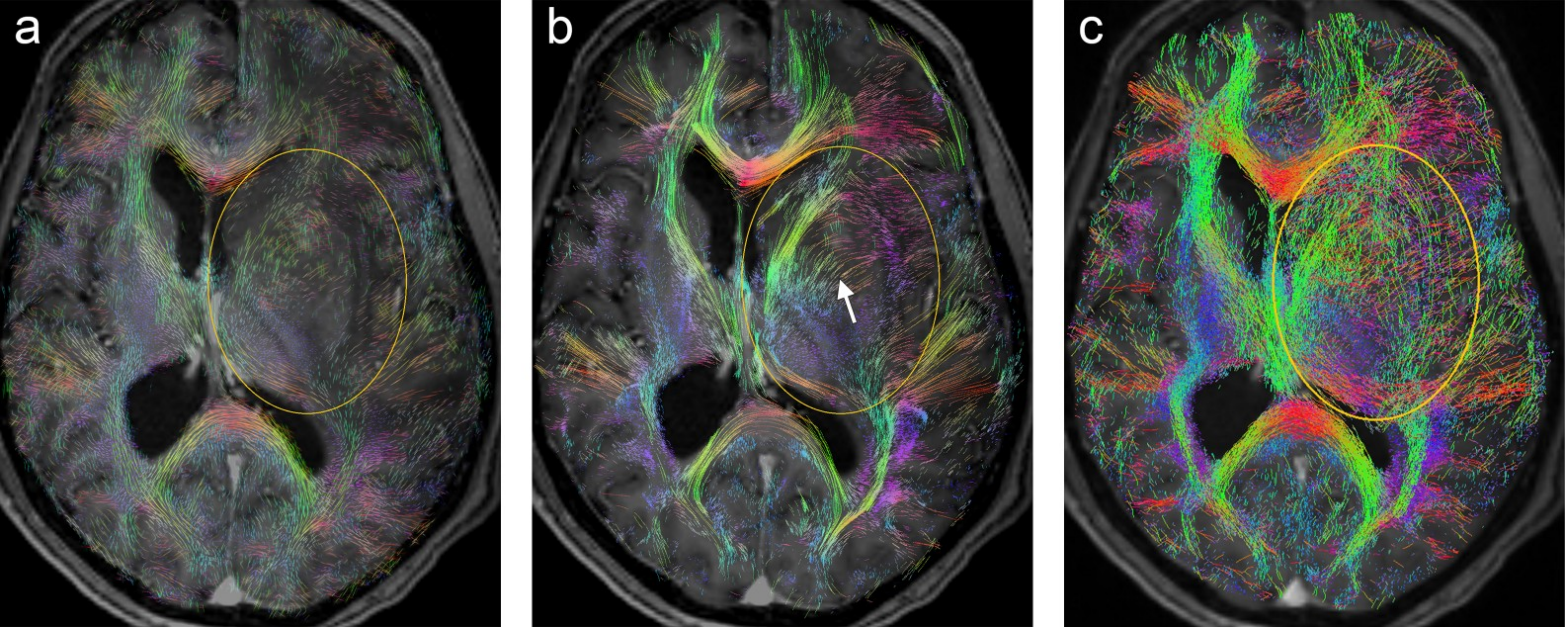
Visualization results from patient E: (a) Transaxial A-Glyph LIC slice with ROI (yellow), and corresponding slices from deterministic (b) and probabilistic (c) tractography results.

**Fig 10:**
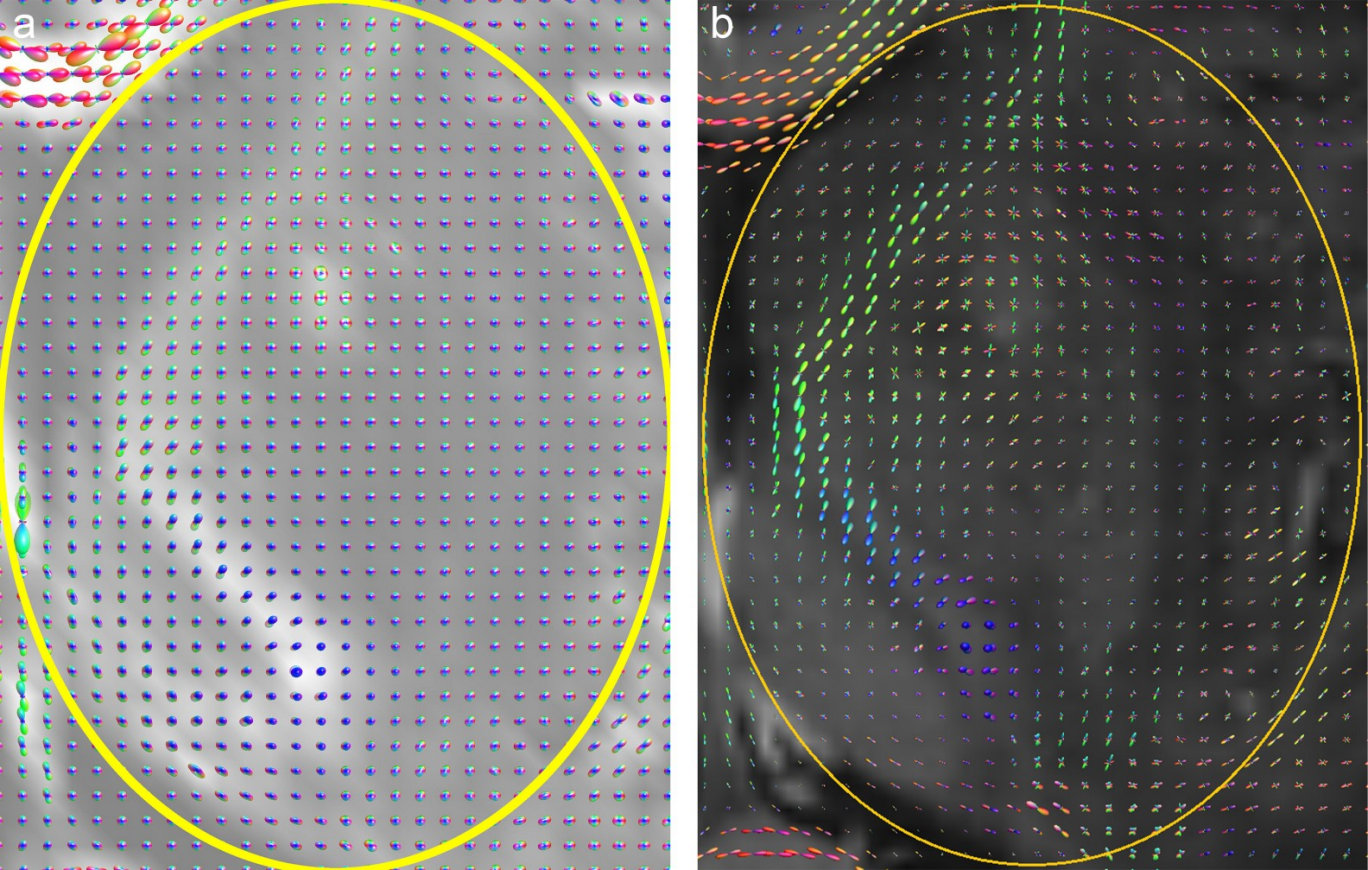
CSA-ODF glyphs (a) and FOD glyphs (b) in ROI of Fig 9 from patient E.

Fig 8 shows T2-weighted (Fig 8A) and T1-weighted (Fig 8B) slice images from patient E, with a region of interest around the peripheral region of a grade IV glioma. As indicated by the primarily spherical shape of ODF glyphs (Fig 10A), the A-Glyph LIC shows a distinct reduction in fiber density, with only a few crossing fibers remaining (Fig 9A). However, deterministic fiber tractography depicts a bunch of dominant fibers without crossings within the tumor region (white arrow in Fig 9B), whereas the probabilistic tractography does not visualize any reduced anisotropy at all (Fig 9C). This effect is obviously caused by the structure of the FODs used by the probabilistic tractography software. Their tendency to sharpen diffusion profiles sometimes leads to an overestimation of local anisotropy, which is seen in the present case (Fig 10B).

**Fig 11:**
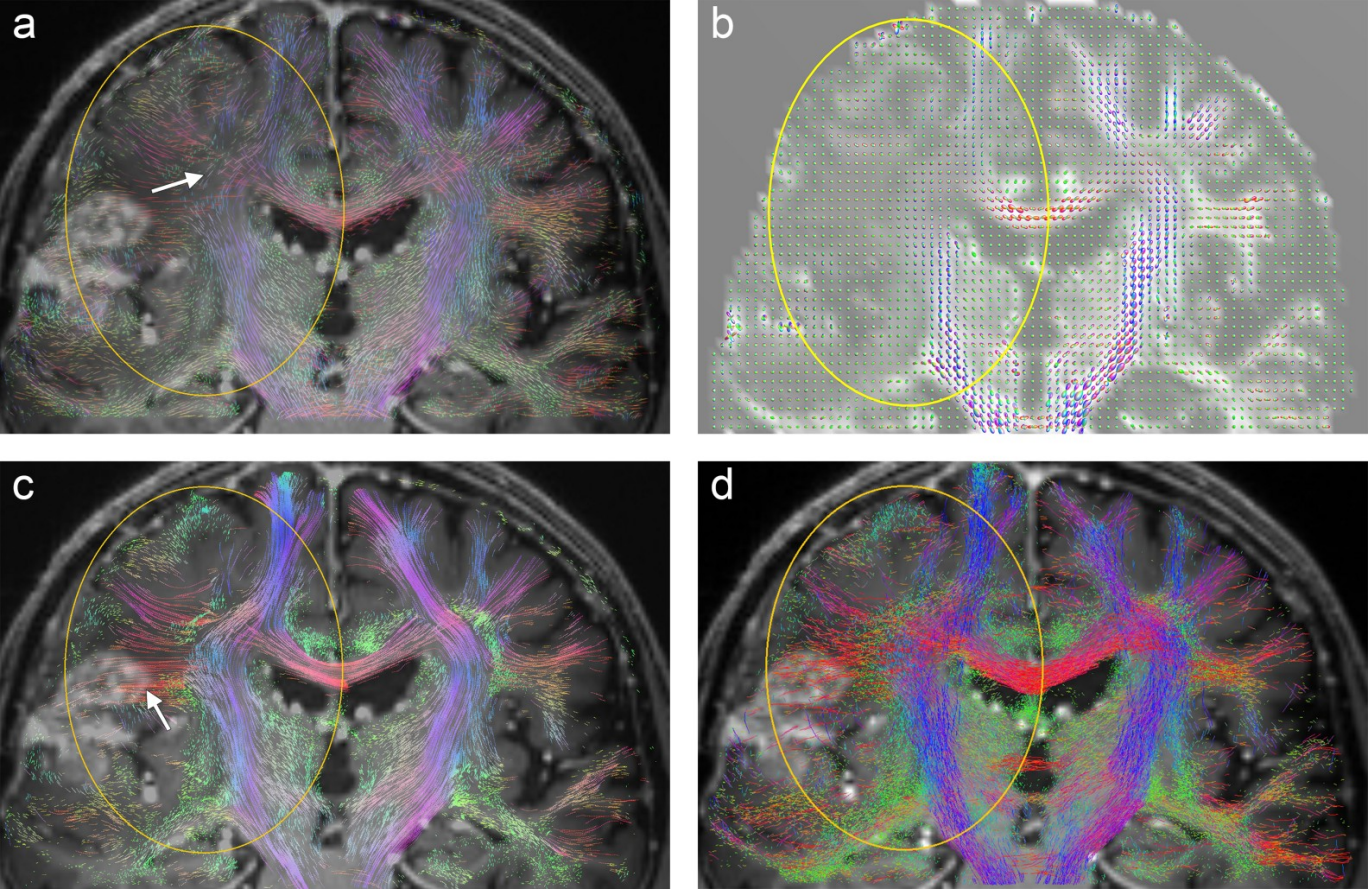
**Visualization results from patient F (coronal slices) with region of interest (yellow): (a) A-Glyph LIC (b) CSA-ODF glyphs, deterministic (c) and probabilistic (d) tractography.** In (c) a bunch of dominant fibers is visualized (arrow) which cannot be explained by the local anisotropies.

In the case of glioma patient F, we see a distinct decay in diffusion anisotropy near the crossing of callosal projections with pyramidal fibers in the right hemisphere. which is clearly depicted by the CSA-ODF glyphs (Fig 11B). This is consistently visualized by the coronal slice image from the A-Glyph LIC volume (arrow in Fig 11A) and slightly less distinctly by the probabilistic tractography result (Fig 11D). Fig 11C shows the corresponding slice from deterministic fiber tractography. Compared to the left hemisphere, a transformation of fiber structures near the crossing area is revealed. However, several dominant fibers are visualized (white arrow) which cannot be explained by the diffusion profiles, represented by the CSA-ODF glyphs.

## 4. Discussion and conclusion

This paper describes a clinical evaluation study of six glioma patients, comparing three fundamentally different fiber visualization algorithms. For all three, a whole-brain seeding strategy was applied to avoid any dependency on the user's skill in correctly defining seed regions and to allow for reproducibility of the visualization results [11,14,46]. Avoiding parameter tuning, a fixed parameter set was applied for each method, independent of the anatomic region affected. Our results show that the three algorithms often lead to different visualizations, indicating different fiber affections. Due to the small sample size, our study is far from providing a thorough clinical evaluation. Its goal was to estimate the potential of techniques apart from tractography or simple metrics-based visualizations like FA/GFA maps, and to disclose preliminary results from applying a connectivity mapping method to pre-operative glioma assessment.

**Fig 12:**
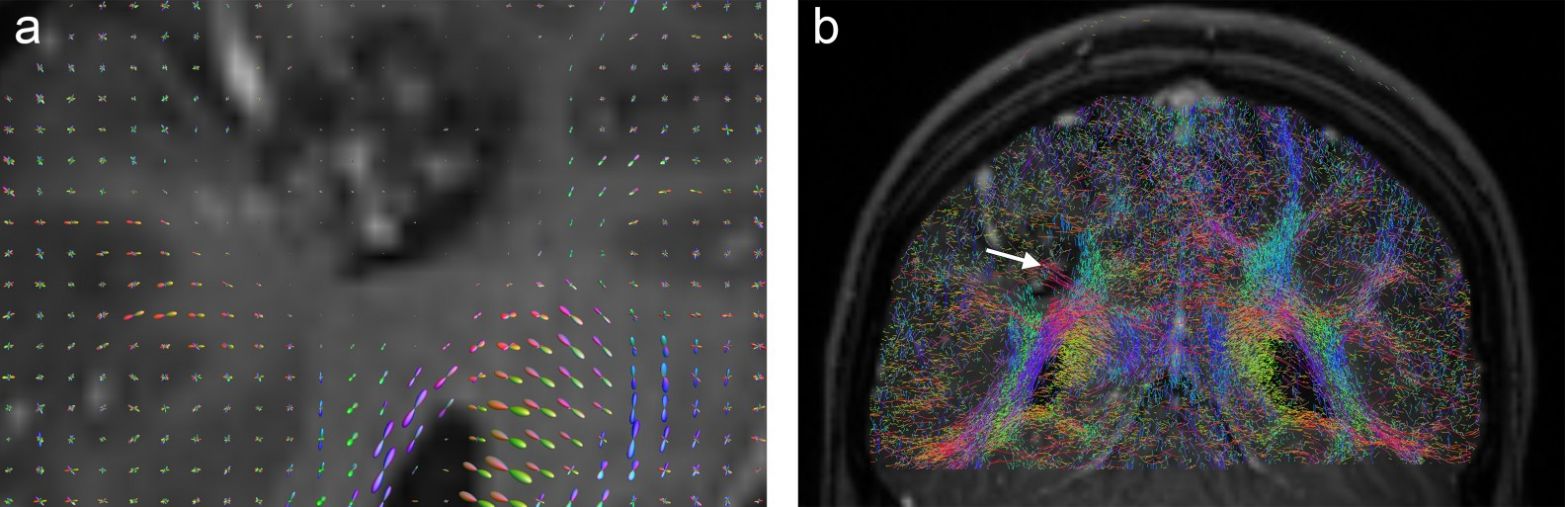
Coronal slices from patient B: (a) FODs and (b) results of probabilistic tractography with minimum FOD length = 0.025, showing erroneous fibers within the cavernoma (white arrow).

In the clinical cases investigated here, deterministic tractography produced strikingly smooth fibers and suffered from problems with false positive tracts (see Figs 1C, 5C, 9B and 11C). In some cases, the method was also unable to correctly depict crossing fiber pathways (see Fig 11C). Probabilistic tractography also tended to produce false positive fibers (see Figs 5C and 9C), which is consistent with previous studies [8,47]. This might be due to an over-sharpness of the FODs used for anisotropy evaluation and tract direction computation. In one case, there was disruption of tracts near the tumor (see Fig 4D). The constrained spherical deconvolution approach does not lead to fully normalized FODs, but uses a model of a single-fiber white matter population, estimated from the individual patient dataset. The automatic definition and adaptation of this model to an individual diffusion dataset [5] may not always lead to perfect results. In the case under consideration, the computed FODs in a circular belt surrounding the tumor were unrealistically small (Fig 12A). Only by diminishing the minimum FOD length parameter, which was used as the tractography stopping criterion, from 0.1 to 0.025 was it possible to visualize fibers in the immediate vicinity of the lesion. However, this remedy has side-effects, producing fibers which are not expected in the central part of the cavernoma (Fig 12B, white arrow). Using CSA-ODFs instead of FODs for probabilistic tractography might solve this problem, but MRtrix does not include CSA-ODF computation and it provides no import function, allowing usage of CSA-ODFs calculated with other software tools. However, MRtrix is a widely used and powerful toolset for probabilistic tractography and therefore is suitable for a clinical study like ours.

The A-Glyph LIC approach produced results that were consistent with the local anisotropy profiles and the findings from the morphologic MR images in most cases. Sometimes, the method tends to disguise fibers running orthogonally to the plane (see Fig 2B). These fiber structures could be better visualized by generating orthogonal planes or zoomed ROIs.

In patient studies with DW-MRI, not knowing the ground truth is one of the main problems confronting researchers. A possible solution, proposed by multiple authors, is checking fiber integrity with intraoperative direct electrical stimulation (DES) [12,48], which seems to be the gold standard for intraoperative identification of eloquent structures. However, the method suffers from various limitations. Firstly, the estimation of fiber location from DES is difficult and subject to a high variability, particularly when using bipolar stimulation [8]. Secondly, brain shift and the shift of white matter pathways near the resection cavity limits the comparison of preoperatively reconstructed fiber tracts to intraoperatively registered stimulation points [49,50]. Stadlbauer et al. propose using MR spectroscopic imaging to measure metabolite concentrations for choline-containing compounds, creatine and N-acetylaspartate to rate fiber integrity [17]. Another method is the combined use of functional MRI (fMRI) and DW-MRI to analyze white matter affection by tumor growth [51,52]. The clinical study presented in this paper does not intend to evaluate any neuroimaging method as a whole, including data acquisition, preprocessing, computation of local diffusion profiles and visualization. Instead, the study focusses on the visualization step of the process only. It pursues the objective of analyzing how local diffusion profiles are depicted as fiber patterns by three fundamentally different visualization methods. Visualization outcomes were verified by analyzing local diffusion profiles, represented by CSA-ODFs, which are free of modeling assumptions. Additionally, morphologic MRI data were used to evaluate the results. Since our clinical study is based on a specified data acquisition and preprocessing protocol, care has to be taken to transfer our findings to other clinical scenarios with different diffusion MRI acquisition and diffusion profile computation methodologies. However, our findings are widely consistent with other studies described in the scientific literature. It confirms the superiority of probabilistic over deterministic tractography for preoperative tumor assessment [12]. It also confirms prior findings that the probabilistic approach tends to produce false positive fibers and inherently requires parameter fine-tuning to track through tissue with different grades of affections, e.g., through edema [14].

The most important original aspect of our approach for glioma assessment by diffusion MRI is its consideration of a non-tractography method for fiber visualization. In pre-surgical diffusion MRI, most clinical studies have been dedicated to the evaluation of all kinds of tractography approaches, e.g., DTI-based tractography with a single-tensor model, HARDI-based deterministic or probabilistic tractography, or tractography with a two-tensor model. However, fiber tracking over longer distances is prone to accumulating errors, e.g., in selecting the correct local tracking direction. Stopping criteria, such as FA or GFA thresholds, must be fine-tuned. For example, seed and exclusion regions have to be defined to allow tracking through edematous regions. It has been demonstrated that by applying sophisticated tractography methods such as FOD-based probabilistic or Kalman filter tractography [13,14], good results may be achieved in pre-operative tumor assessment. However, profound algorithmic knowledge and a great deal of experience in the application of these methods, combined with the fine-tuning of parameters, is necessary to avoid mistakes. This has hampered their clinical practicality and prevented their widespread usage in clinical routine.

Particularly in situations where local affection of fiber pathways rather than global connectivity is the center of attention, it might be useful to consider visualization methods other than tractography. Visualization of merely local diffusion features, e.g., as color-coded FA or GFA maps, or as ODF or FOD glyphs, makes it difficult to evaluate local connectivity, recognize fiber patterns and assess fiber affections. Therefore, visualization methods providing some kind of local connectedness by aggregating information from a local neighborhood seem to be more promising. Some applications of such techniques to diffusion MRI have been published in the scientific literature. Apart from the A-Glyph LIC method used in this paper, further examples are dense ellipses [53], glyph packing [54] and merging ellipsoids [55]. These have in common that they compute some kind of local diffusion coherence measure between neighboring voxels and thus are not only able to depict local diffusion profiles, but also allow for the visualization of fiber patterns. This makes assessment of fiber affection possible. Since they do not include far-reaching data interpretations, they are not so prone to errors and do not suffer from the problem of error propagation. Unfortunately, most of these methods have not yet been evaluated clinically.

One of the main advantages of tractography methods is their capability of easy 3D visualization, for example by streamlines or streamtubes. This allows the assessment of three-dimensional spatial relationships, e.g., between a white matter lesion and adjacent fiber bundles. In our clinical study, we used two-dimensional slice images to reveal fiber structures. Slice images are good for providing a detailed view and allow easy fusion with T1- or T2-weighted MRI images. On the other hand, they make it hard for a neurosurgeon to perceive the three-dimensional reality. Volume visualization of line integral convolution datasets suffers from the problems of occlusion and the superimposition of multiple fiber pathways, which disguise relevant information. To tackle this problem, the scientific literature focuses on masking structures which, in a particular clinical situation, are not of so much interest. Tax et al. propose making fiber trajectories that are oriented along a user-specified opacity axis transparent [56]. Another approach would be to define anatomic volumes of interest (VOIs) and make fiber structures outside of them more transparent. Such VOIs could easily be delineated using an anatomic brain atlas matched to the individual patient dataset [16].

Our results from comparing streamline tractography to a non-tractography approach suggest that pursuing practical methods which visualize diffusion features and fiber patterns by aggregating signal information from a local neighborhood, rather than trying to provide far-reaching data interpretations, holds potential for further research and might open up new perspectives for the clinical application of DW-MRI.
